# Language profiles and literacy outcomes of children with resolving, emerging, or persisting language impairments

**DOI:** 10.1111/jcpp.12497

**Published:** 2015-12-17

**Authors:** Margaret J. Snowling, Fiona J. Duff, Hannah M. Nash, Charles Hulme

**Affiliations:** ^1^ Department of Experimental Psychology and St. John's CollegeUniversity of OxfordOxfordUK; ^2^ School of PsychologyUniversity of LeedsLeedsUK; ^3^ Division of Psychology and Language SciencesUniversity College LondonLondonUK

**Keywords:** Language disorder, reading, language, specific language impairment

## Abstract

**Background:**

Children with language impairment (LI) show heterogeneity in development. We tracked children from pre‐school to middle childhood to characterize three developmental trajectories: resolving, persisting and emerging LI.

**Methods:**

We analyzed data from children identified as having preschool LI, or being at family risk of dyslexia, together with typically developing controls at three time points: t1 (age 3;09), t3 (5;08) and t5 (8;01). Language measures are reported at t1, t3 and t5, and literacy abilities at t3 and t5. A research diagnosis of LI (irrespective of recruitment group) was validated at t1 by a composite language score derived from measures of receptive and expressive grammar and vocabulary; a score falling 1*SD* below the mean of the typical language group on comparable measures at t3 and t5 was used to determine whether a child had LI at later time points and then to classify LIs as resolving, persisting or emerging.

**Results:**

Persisting preschool LIs were more severe and pervasive than resolving LIs. Language and literacy outcomes were relatively poor for those with persisting LI, and relatively good for those with resolving LI. A significant proportion of children with average language abilities in preschool had LIs that emerged in middle childhood – a high proportion of these children were at family risk of dyslexia. There were more boys in the persisting and resolving LI groups. Children with early LIs which resolved by the start of formal literacy instruction tended to have good literacy outcomes; children with late‐emerging difficulties that persisted developed reading difficulties.

**Conclusions:**

Children with late‐emerging LI are relatively common and are hard to detect in the preschool years. Our findings show that children whose LIs persist to the point of formal literacy instruction frequently experience reading difficulties.

## Introduction

Language impairment (LI) is a relatively common developmental disorder (Tomblin et al., 1997) which can occur alongside low nonverbal ability (nonspecific language impairment, NLI), or average nonverbal ability (specific language impairment, SLI; Hayiou‐Thomas, Oliver, & Plomin, 2005). Moreover, there is comorbidity between LI and reading disorder/dyslexia (McArthur, Hogben, Edwards, Heath, & Mengler, 2000). Classification of LI, however, is not stable and rates of persistence vary from 11%–92% (Tomblin, Zhang, Buckwalter, & O'Brien, 2003). Particularly relevant are the findings of Bishop and colleagues, who followed a clinically referred sample of children at ages 4, 5½, 8½, and 15½ years (Bishop & Adams, 1990; Bishop & Edmundson, 1987; Snowling, Bishop, & Stothard, 2000; Stothard, Snowling, Bishop, Chipchase, & Kaplan, 1998). Of those with SLI at age 4, 53% had persisting LI at 5½; and 56% at 8½ (see also Cole, Schwartz, Notari, Dale, & Mills, 1995). When followed up in adolescence, 90% of those with SLI persisting to 5½ years had persistent SLI or NLI (Stothard et al., 1998). Importantly, 35% of those who appeared to have resolved their LI by 5½ years had relapsed in adolescence, raising the issue of possible ‘illusory’ recovery (Scarborough & Dobrich, 1990). Reading and spelling skills were within the normal range for children whose LIs had resolved by 5½ but poor in those with persisting LI, particularly if performance IQ was below average (Snowling et al., 2000).

Resolving and persisting trajectories are not the only patterns of development observed among children with language difficulties. Studies of early language delay (ELD or ‘late‐talkers’) have also revealed a late‐onset or emerging trajectory (e.g., Dale, Price, Bishop, & Plomin, 2003). Henrichs et al. (2011), following infants from 1½ to 2½ years, and Zambrana, Pons, Eadie, and Ystrom (2014), following children from 3 to 5 years, reported similar prevalence rates of 3% for persisting LI, 5–6% for resolving LI, and 6% for emerging LI. Examining predictors of the different patterns, Zambrana et al. (2014) reported gender differences with boys being most common in the persistent group, less common in the transient group and least common in the emerging group. Persistent LI was associated with a range of risk factors including social adversities. Interestingly, they also reported an association between the late‐onset trajectory and a family history of literacy problems. As preschool phonological impairments are common in children at family risk of dyslexia (e.g., Nash, Hulme, Gooch, & Snowling, 2013), such deficits may have downstream effects not only on the development of reading but also, as suggested in a phonological theory of SLI proposed by Chiat (2001), on lexical and syntactic development.

A limitation of current findings regarding the trajectories of LI is that data come primarily from parental report measures. In addition, while the focus has been on early language milestones, a key issue is how these children's literacy skills develop. It is well documented that children with LI typically experience literacy difficulties (e.g., Catts, Adlof, Hogan, & Ellis Weismer, 2005) but the developmental relationship between early LI and later literacy difficulties is not straightforward (Bishop & Snowling, 2004). First, according to the ‘critical age hypothesis’, it is only when language difficulties are present *at the point of reading instruction* that they have detrimental effects on reading development. Bishop and colleagues initially considered that the impact was via expressive phonological difficulties, but later implicated broader aspects of language (Bishop & Adams, 1990; Stothard et al., 1998). Second, studies of children at family risk of dyslexia have revealed not only that early oral language difficulties often presage poor literacy but also that their effects are mediated by poor phonological awareness (e.g. Torppa, Lyytinen, Erskine, Eklund, & Lyytinen, 2010). Third, the association reported by Zambrana et al. (2014) between an emerging LI trajectory and family risk of literacy problems highlights a further possibility – that some children with preschool phonological impairments associated with family risk of dyslexia may in addition experience downstream effects of these deficits on broader oral language skills.

The main aim of the present study was to use objective measures of language and literacy to validate previous findings regarding resolving, emerging, and persisting trajectories of LI. Given the well‐established finding that preschool language impairments are a risk factor for dyslexia (Snowling & Melby‐Lervag, 2016; for a review) we assessed children at high risk of dyslexia either because they were at familial risk or because they had a preschool LI. The children were followed from 3½ through 5½ to 8 years. A particular focus here is on children with late‐emerging LI. Based on previous research, we hypothesized that the persisting and resolving trajectories would be more common among boys than girls, that the emerging trajectory would be strongly associated with family risk of dyslexia and that the persisting trajectory would be associated with a great number of adversities including social disadvantage (Hoff, 2006 for discussion). In terms of literacy outcomes, we predicted that children who showed resolving language impairment would be least at risk of reading and spelling problems, while children with emerging and persisting impairments would be more severely affected, consistent with their poorer language skills at the time of formal reading instruction.

## Method

### Participants

Children participating in this study were part of the Wellcome Language and Reading Project. Ethical permission for the project was granted by the Psychology Department, University of York, and the NHS Research Ethics Committee. Informed written consent was obtained from the children's parents. Children were recruited because they were at risk of developing dyslexia (owing to a family risk (FR), or because parents were concerned they had a preschool language impairment (LI)) or because they were typically developing with typical language development (TL). Children were categorized as FR if they had a parent or sibling who could be classified as dyslexic (see Nash et al., 2013, for further details).

The children were assessed at six time points: t1 (age 3½), t2 (age 4½), t3 (age 5½), t4 (age 6½), t5 (age 8), and t6 (age 9). Here, we report data relating to 220 children for whom we have language measures over three time points: t1 (mean age = 3;09, *SD *= 0;03), t3 (5;08 (0;03)), and t5 (8;01 (0;06); complete data for *n* = 184) and literacy‐related measures at t3 and t5 (complete data for *n* = 213).

#### Language impairment

At each time point, criteria were applied to determine which children could be classified as language impaired. Following recruitment, children were classified as LI or not according to the conventions normally used (see below). However, because we wished to investigate novel categories of language impairment, it was important to ensure reliability of measurement (in particular to guard against regression to the mean which could be said to characterize the ‘resolving’ group). We therefore followed a procedure which defines LI as falling – 1 SD below the mean of a composite language measure at t1, t3, t5. The composite measure contained the same constructs at each time point: expressive vocabulary, receptive grammar and expressive grammar. There were identical tests at t1 and t3; by necessity, two of the three specific measures were different at t5 (see details of tests used to form composites below). LI classification was made without regard to nonverbal ability.

#### Language impairment at t1

Children were classified as LI if they ‘failed’ at least 2/4 language tests. Three subtests were from the *Clinical Evaluation of Language Fundamentals were used* (*CELF‐Preschool 2 UK* – Wiig, Secord, & Semel, 2006): *Basic Concepts, Expressive Vocabulary, Sentence Structure*; and a scaled score of 7 or below counted as a fail. For the *Test of Early Grammatical Impairment* (*TEGI* – Rice & Wexler, 2001), failure of the screener constituted a fail (see Nash et al., 2013). For this study, we validated this clinical classification at t1 using scores on the composite language measure (*Expressive Vocabulary, Sentence Structure, TEGI, op cit)*. All but two children who were clinically classified also fulfilled the criterion of falling −1 SD below the mean of a composite language measure for the whole sample. These two cases were dropped so that all cases at t1 in this paper fulfilled the dual criteria of (i) clinical classification (ii) 1 SD below the mean on a reliable composite language measure. Given that children with language difficulties are heavily overrepresented in this sample, this criterion of −1 SD below the total sample mean can be regarded as quite a conservative definition of LI.

#### Language impairment at t3 and t5

A child was classified as LI if their score fell 1 SD below the mean of a composite measure t3 – *Expressive Vocabulary, Sentence Structure, TEGI, op cit*; t5 – *Expressive Vocabulary (op cit), Test for Reception of Grammar* (*TROG‐II* – Bishop, 2003) and *Formulated Sentences* (*CELF 4*).

#### Language trajectories

LI status at t1, t3, and t5 was used to characterize each child's language trajectory. Children with typical language (TL) were those who never reached LI classification. ‘Resolving’ LI applied to those who had LI at t1 and/or t3, but not t5; ‘emerging’ LI to those who had no LI at t1, but LI at t5 (regardless of t3 status); and ‘persisting’ LI to those who had LI at t1 and t5 (regardless of t3 status). We placed more emphasis on t1 (the age of first ‘diagnosis’) and t5 when we expected language development to have stabilized. Data from t3 allow us to assess the ‘critical age’ hypothesis. It should be noted that we did not use any measures of phonological processing in setting the criteria for LI or for defining language trajectory.

### Tests and procedures

Children were assessed individually by a member of the research team at each time point (t1, t3, t5), at home or school. The tests were administered as part of a larger assessment session, lasting approximately 1½–2 hr per child (with appropriate rest breaks given). The following standardized tests were administered as per instructions in the manuals (more details of tests can be found in Nash et al., 2013 and Thompson et al., 2015).

#### Nonverbal ability

At t1, nonverbal ability was measured using two subtests from the *Wechsler Preschool and Primary Scale of Intelligence* (*WPPSI‐III* – Wechsler, 2003a): *Block Design* and *Object Assembly*. At t5, two subtests from the *Wechsler Intelligence Scale for Children* (*WISC‐IV* – Wechsler, 2003b) were used: *Block Design* and *Matrices*. At both time points, composite nonverbal IQ scores were calculated based on the mean of z‐standardized scores for the two subtests; the average standard scores based on the subtests were also calculated.

### Language‐related measures

#### Vocabulary

Expressive vocabulary was assessed using the *Expressive Vocabulary* subtest at t1 (*CELF‐Preschool 2 UK* – Wiig et al., 2006), t3 and t5 (*CELF 4* – Semel, Wiig, & Secord, 2003). At t5, the test was extended with eight items from the *Expressive One Word Picture Vocabulary Test* (Brownell, 2000) to guard against ceiling effects.

Receptive language was assessed at t1 using the *Basic Concepts* subtest (*CELF‐Preschool 2 UK*), and at t5 with the *Receptive One Word Picture Vocabulary Test* (Brownell, 2000).

#### Grammar

At t1 and t3, receptive grammar was assessed using the *Sentence Structure* subtest (*CELF 4*). Inflectional morphology was measured via two subtests of the *TEGI* (Rice & Wexler, 2001): the third person and past tense probes. The percentage of correct responses across both probes is reported.

At t5, receptive grammar was assessed using the *Test for Reception of Grammar* (*TROG‐II* – Bishop, 2003); we report the number of blocks successfully completed (4 items per block). Expressive grammar was measured using the *Formulated Sentences* subtest (*CELF 4*).

### Literacy‐related measures

#### Letter‐sound knowledge

At t3, the extended version of the *Letter Knowledge* subtest from the *York Assessment for Reading Comprehension* (*YARC* – Hulme et al., 2009) was administered.

#### Phoneme awareness

At t3 and t5, the *Phoneme Deletion* subtest from the *YARC* (Hulme et al., 2009) was administered. At t5, 12 items were added to extend the test (five words with picture support and seven nonwords without picture support) to guard against ceiling effects.

### Rapid automatized naming

Rapid automatized naming (RAN) was measured at t3 and t5: children named aloud a series of five objects repeated at random in an 8 by 5 matrix. *RAN Objects* was indexed by a rate score – number of objects correctly named per second.

### Word‐level literacy

The *Single Word Reading Test* (*SWRT* – Foster, 2007) was used to assess word reading accuracy at t3 and t5.

Nonword reading was assessed at t5 by the *Graded Nonword Reading Test* (*GNWRT* – Snowling, Stothard, & McLean, 1996).

The *Spelling* subtest of the *Wechsler Individual Assessment Test* (*WIAT‐II* – Wechsler, 2005) was administered at t5.

### Prose reading

The *Passage Reading* subtest of the *York Assessment for Reading Comprehension* (Snowling et al., 2009) was administered at t5. Children scoring <30 on the *SWRT* began at Beginner Level; children scoring 30 on the *SWRT* began at Level 3. They read the passages aloud in their own time and then answered questions about them. *Reading Comprehension* is the total number of comprehension questions answered correctly. Raw scores were based on all seven passages, with full credit awarded for passages below basal and maximum errors assumed for passages above ceiling. Standard scores were based on the two highest passages (as per the manual).

## Results

Our sample focused on dyslexia and purposefully oversampled for LI. Table 1 shows the language composite scores (with 95% confidence intervals) derived from measures of expressive language, receptive grammar, and expressive grammar at the three time points (t1, t3, t5) for each language trajectory group. At all time points, the TL groups are significantly different from each of the other groups and there is no overlap in the confidence intervals. At t1, the resolving and persisting LI groups are not significantly different as confirmed by the overlapping confidence intervals; moreover, these do not overlap with those of the emerging group. At t5, the confidence intervals for the resolving and persisting LI groups no longer overlap but those of the emerging and persisting LI groups do (neither show overlap with those of the resolving group). At t3, each of the groups differs from the others; there is a step function such that scores for TL > resolving > emerging > persisting and there is no overlap between the confidence intervals around the means for each group.

**Table 1:** Mean language composite scores for the typical language, resolving LI, emerging LI, and persisting LI trajectories (*Z* scores with 95% confidence intervals)

	Language composite t1			Language composite t3			Language composite t5		
	Mean	*SD*	95% CI	Mean	*SD*	95% CI	Mean	*SD*	95% CI
TL (*N* = 145)	0.47	0.60	.37 to 0.57	0.41	0.57	.32 to .51	0.51	0.48	0.43 to 0.58
Resolving (*N* = 12)	−0.89	0.36	−1.12 to −.66	−0.05	0.35	−.28 to .18	0.04	0.34	−0.18 to 0.25
Emerging (*N* = 21)	−0.33	0.37	−.50 to −.17	−0.56	0.55	−.81 to −.31	−1.12	0.53	−1.36 to −0.88
Persisting (*N* = 42)	−1.08	0.52	−1.25 to −.9	−1.04	0.59	−1.22 to −.86	−1.23	0.44	−1.37 to −1.09

Table 2 shows the age, SES, gender and family risk (FR) status of each language trajectory, along with information regarding the percentage of each group who had speech difficulties at recruitment. Of the sample, 34% (*n* = 75) were LI at some point. The remaining children were considered to have typical language (TL). Of the LIs present at t1, 22% had resolved and 78% were persisting at t5. Using data from t1, t3, and t5 to describe trajectories of LI, 16% could be classed as resolving, 28% emerging, and 56% persisting. If a more stringent cut‐off of −1.5 SD below the mean is taken to classify a child as LI, 6/42 children from the persisting group and 7/12 children from the resolving group no longer fulfill criteria for LI at t1. However, all of the children in the persisting group fulfill this strict criterion at t5. It seems therefore that using a more conservative criterion reduces the false positive rate (i.e. misclassifying as LI at time 1 children who go on to be free of LI ~4 years later) such that only 2% of the sample now show this profile, but it has little effect on those whose language impairments persist. Using the stricter criterion at t5 reduces the number in the emerging group from 21 to 16 making up 7.3% of the sample.

**Table 2:** Age, gender, family risk for dyslexia status (FR), and percentage with SSD for the four language trajectories

Trajectory	*N*	Age t1 (*SD*)	Age t3 (*SD*)	Age t5 (*SD*)	% males	% FR dyslexia	% speech difficulties	SES
TL	145 (66%)	3;09 (0;04)	5;08 (0;03)	8;03 (0;04)	54	46	14	.24^a^ (.66)
Resolving	12 (6%)	3;08 (0;03)	5;08 (0;02)	8;02 (0;03)	75	50	42	.05^a^ (.70)
Emerging	21 (10%)	3;08 (0;03)	5;07 (0;03)	7;09 (0;05)	48	76	48	−.06^a^ (.79)
Persisting	42 (19%)	3;08 (0;02)	5;07 (0;03)	7;11 (0;05)	79	48	57	−.32 (.84)

For SES, values with the same superscript do not differ significantly (*p* > .05).

Gender was associated with LI trajectory (*χ* ^*2*^(3) = 10.68, *p *=* *.014) with more boys in the persisting than the TL group (OR = 3.15, *p* < .005). Importantly, although there was no significant overall association between FR status and LI trajectory (*χ* ^*2*^(3) = 6.67, *p *=* *.083), there were more children at family risk of dyslexia in the emerging than the TL group (OR = 3.80, *p* < .014).

### Characterization of language trajectories

Raw scores for language and nonverbal abilities are given in Table 3. The standard scores in Table 4 (calculated where appropriate) indicate how the groups are performing relative to age norms.

**Table 3:** Raw scores for all nonverbal and language measures, with effect sizes for between‐group contrasts

Language/nonverbal measure (maximum score)	Reliability	Language trajectory	Mean (*SD*)	Effect sizes (*d*) for group contrasts		
				Resolving	Emerging	Persisting
Nonverbal IQ^a^ t1 (n/a)	.84^d^	TL	112.5 (14.5)	.86*	.77*	0.96*
		Resolving	100.5 (6.25)	–	0.14	0.53
		Emerging	101.71 (10.07)		–	0.23
		Persisting	98.92 (13.27)			–
Basic concepts t1 (18)	.90^d^	TL	16.03 (1.78)	2.03*	1.07*	2.75*
		Resolving	12.25 (2.80)	–	0.89	0.22
		Emerging	14.14 (1.80)		–	1.46*
		Persisting	10.20 (3.10)			–
Expressive vocabulary t1 (40)	.88^d^	TL	20.07 (5.36)	1.26*	0.93*	2.17*
		Resolving	13.25 (6.73)	–	0.40	0.39
		Emerging	15.24 (3.91)		–	1.46*
		Persisting	8.74 (4.81)			–
Receptive grammar t1 (22)	.77^d^	TL	14.18 (3.00)	2.59*	1.00*	1.98*
		Resolving	6.50 (2.80)	–	1.66*	0.73
		Emerging	11.19 (2.96)		–	0.95
		Persisting	7.98 (3.66)			–
Inflectional morphology^b^ t1 (n/a)	.87^e^	TL	74.30 (23.80)	1.46*	0.84*	2.04*
		Resolving	39.66 (24.82)	–	0.52	0.03
		Emerging	53.68 (29.45)		–	1.08
		Persisting	26.50 (22.00)			–
Expressive vocabulary t3 (54)	.91^d^	TL	31.04 (6.58)	1.00*	1.53*	2.14*
		Resolving	24.58 (4.91)	–	0.61	0.99*
		Emerging	21.05 (6.43)		–	0.62
		Persisting	17.29 (6.05)			–
Receptive grammar t3 (26)	.74^d^	TL	21. 97 (2.52)	0.72	1.27*	1.97*
		Resolving	20.17 (2.62)	–	0.49	0.82*
		Emerging	18.57 (3.75)		–	0.60
		Persisting	16.19 (4.13)			–
Inflectional morphology^b^ t3 (n/a)	.87^e^	TL	96.88 (6.60)	0.71	0.85	1.56*
		Resolving	92.04 (9.77)	–	0.19	0.53*
		Emerging	88.78 (20.83)		–	0.57*
		Persisting	73.90 (28.76)			–
Nonverbal IQ^c^ t5 (n/a)	.84^e^	TL	0.34 (0.76)	0.43	1.42*	1.55*
		Resolving	0.02 (0.55)	–	1.25	1.25*
		Emerging	−0.73 (0.63)		–	0.15
		Persisting	−0.82 (0.71)			–
Receptive vocabulary t5 (170)	.78^d^	TL	100.63 (11.44)	0.50	1.53*	1.73*
		Resolving	95.00 (9.32)	–	1.17*	1.27**
		Emerging	83.43 (10.59)		–	0.21
		Persisting	81.19 (10.63)			–
Expressive vocabulary t5 (70)	.87^d^	TL	49.06 (6.28)	0.85	2.31*	2.53*
		Resolving	43.04 (3.26)	–	1.61*	1.47*
		Emerging	34.57 (5.60)		–	0.35
		Persisting	32.33 (7.24)			–
Receptive grammar t5 (20)	.88^f^	TL	15.69 (2.22)	0.50	3.03*	2.85*
		Resolving	14.58 (2.02)	–	2.15*	1.98*
		Emerging	7.33 (3.30)		–	0.53
		Persisting	8.59 (3.18)			–
Expressive grammar t5 (56)	.76^d^	TL	40.01 (7.34)	0.48	1.73*	2.09*
		Resolving	36.50 (7.04)	–	1.26*	1.40*
		Emerging	27.29 (7.80)		–	0.38
		Persisting	24.19 (8.52)			–

a Performance IQ.

b Total percent correct.

c Composite *z* score.

d Test/retest reliability.

e Average test/retest reliability across two subtests.

f Internal consistency.

*p <* *.05; **p <* *.01; ***p <* *.001.

**Table 4:** Standardized nonverbal and language test scores, for the four language trajectories

Language/nonverbal measure	TL	Resolving	Emerging	Persisting
Nonverbal IQ^a^ t1	112.46 (14.47)	100.50 (6.24)	101.71 (10.07)	98.92 (13.24)
Basic concepts^b^ t1	11.69 (2.16)	7.92 (2.61)	9.52 (2.27)	6.43 (2.00)
Expressive vocabulary^b^ t1	11.63 (2.20)	8.75 (3.17)	10.10 (1.67)	6.66 (2.60)
Receptive grammar^b^ t1	10.83 (2.26)	5.67 (1.61)	8.95 (1.96)	6.73 (2.42)
Expressive vocabulary^b^ t3	12.80 (2.72)	10.83 (1.90)	9.62 (2.18)	8.43 (1.80)
Receptive grammar^b^ t3	12.21 (2.04)	11.08 (1.98)	9.62 (2.80)	8.05 (2.84)
Nonverbal IQ^a^ t5	108.81 (12.62)	103.25 (10.42)	93.00 (11.08)	88.79 (13.50)
Receptive vocabulary^a^ t5	113.94 (11.51)	106.08 (12.31)	100.05 (10.66)	96.55 (10.71)
Expressive vocabulary^b^ t5	12.90 (2.18)	10.83 (1.70)	8.76 (1.70)	7.90 (2.00)
Receptive grammar^a^ t5	105.23 (10.49)	99.75 (11.09)	75.00 (13.30)	74.83 (13.56)
Expressive grammar^b^ t5	10.48 (2.75)	8.67 (3.17)	6.62 (2.51)	5.33 (2.79)

a Standard score (*M* = 100, *SD *= 15).

b Scaled score (*M* = 10, *SD *= 3).

From the raw scores, the general pattern is for the LI groups to perform below the TL group, but not always similarly to one another. To evaluate the differences in raw scores between groups, Cohen's d was calculated, correcting for unequal sample sizes. We interpret *d *=* *0.8 as a large, *d *=* *0.5 a moderate, and *d *=* *0.3 a small effect size. Asterisks in Table 3 represent significance levels of group comparisons after controlling for multiple comparisons (Bonferroni correction *α *= .004); where appropriate transformations were applied to improve the normality of variables with significantly non‐normal distributions before testing for group differences.

On nonverbal IQ, the LI groups perform similarly to one another at t1 with lower scores than the TL group but the emerging and persisting groups show a substantial decline by t5 to perform lower than the TL and resolving groups. As might be expected, all LI groups perform worse than the TL group on language measures at every time point, with medium to large effect sizes. It is important to note that, at t1, the emerging group always performs better than the resolving group with a large effect size for receptive grammar. However, at t3, this pattern reverses with the emerging group scoring less well than the resolving group on all measures. These deficits in the emerging group increase over time, so that by t5, they are performing worse than the resolving group and similarly to the persisting group on all measures.

At t1, the resolving group (at this time point classified as having LI) performs significantly worse than the TL group on all language measures, with large effect sizes. Standard scores indicate particular difficulties with grammar while expressive vocabulary is in the average range. At t5, the standard scores of the resolving group are comfortably in the average range. Compared to the TL group, the resolving group no longer differs significantly on tests of grammar (though effect sizes are moderate), and the effect size for the difference in expressive vocabulary is large.

Finally, the persisting group shows the most severe difficulties on all language tests at t1 through t5 with standard scores in the below‐average range. Compared with the resolving group at t1, they have lower nonverbal ability (the effect is moderate but not significant) and perform significantly worse on expressive vocabulary and basic concepts (a language comprehension task; although effect sizes are moderate to small). The effect size for receptive grammar is large but favors the persisting group. By t3, the resolving group performs significantly better than the persisting group on all measures, with large effect sizes.

### Literacy outcomes for children with resolving, emerging, and persisting language difficulties

Raw scores for literacy‐related measures are given in Table 5, and standard scores in Table 6. Once again Cohen's d was calculated as a measure of the differences between groups. Asterisks in Table 5 represent the significance levels of group comparisons after controlling for multiple comparisons (Bonferroni correction *α *= .004); where appropriate transformations were applied to improve the normality of variables before testing for group differences.

**Table 5:** Raw scores for all literacy‐related measures, with effect sizes for between‐group contrasts

Literacy‐related measure (maximum score)	Reliability	Language trajectory	Mean (*SD*)	Effect sizes (*d*) for group contrasts		
				Resolving	Emerging	Persisting
Letter‐sound knowledge t3 (32)	.98^b^	TL	30.13 (3.35)	0.11	0.79*	1.14*
		Resolving	30.50 (1.83)	–	0.93	0.82*
		Emerging	27.43 (4.01)		–	0.38
		Persisting	25.17 (6.82)			–
Phoneme deletion t3 (12)	.93^b^	TL	7.25 (2.42)	0.25	0.85*	1.30*
		Resolving	6.67 (1.89)	–	0.66	0.91*
		Emerging	5.19 (2.52)		–	0.44
		Persisting	4.12 (2.46)			–
RAN objects^a^ t3 (n/a)	.75^b^	TL	0.91 (0.18)	0.28	1.11*	0.99*
		Resolving	0.86 (0.18)	–	0.79	0.74
		Emerging	0.70 (0.21)		–	0.16
		Persisting	0.73 (0.17)			–
Single word reading t3 (60)	.98^b^	TL	12.80 (10.16)	0.53	0.93*	1.13*
		Resolving	7.58 (7.76)	–	0.64	0.92
		Emerging	3.90 (4.56)		–	0.32
		Persisting	2.51 (4.26)			–
Phoneme deletion t5 (24)	.93^b^	TL	18.59 (4.76)	0.18	1.14*	0.87*
		Resolving	18.17 (2.98)	–	0.84*	0.64*
		Emerging	12.67 (5.90)		–	0.16
		Persisting	13.45 (5.78)			–
RAN objects^a^ t5 (n/a)	.71^b^	TL	1.14 (0.23)	0.22	0.88*	0.64*
		Resolving	1.19 (0.27)	–	0.99*	0.88
		Emerging	0.93 (0.26)		–	0.11
		Persisting	0.99 (0.22)			–
Nonword reading t5 (20)	.90^c^	TL	15.88 (4.30)	0.17	1.01*	0.87*
		Resolving	16.58 (3.09)	–	0.97	0.32*
		Emerging	11.14 (6.84)		–	0.25
		Persisting	11.81 (5.82)			–
Single word reading t5 (60)	.98^b^	TL	38.98 (9.96)	0.20	1.31*	1.05*
		Resolving	37.00 (7.36)	–	1.10	0.85
		Emerging	25.62 (12.16)		–	0.20
		Persisting	28.05 (12.26)			–
Spelling t5 (53)	.96^c^	TL	26.53 (6.00)	0.10	1.14*	0.98*
		Resolving	25.92 (4.66)	–	1.33	0.99
		Emerging	19.90 (4.69)		–	0.11
		Persisting	20.57 (6.42)			–
Prose reading comprehension t5 (56)	.48–.77^d^	TL	36.83 (10.8)	0.23	1.58*	1.51*
		Resolving	34.42 (7.81)	–	1.58*	1.44*
		Emerging	19.95 (10.27)		–	0.09
		Persisting	20.81 (10.13)			–

a Rate score – number of items correct per second.

b Internal consistency.

c Test/retest reliability.

d Internal consistency across all passages.

*p <* *.05; **p <* *.01; ***p <* *.001.

**Table 6:** Standardized literacy‐related scores (*M* = 100, *SD* = 15), for the four language trajectories

Literacy‐related measure	TL	Resolving	Emerging	Persisting
Letter–sound knowledge t3	114.31 (11.81)	114.83 (11.75)	104.14 (11.67)	99.98 (15.11)
Phoneme deletion t3	109.78 (12.01)	107.67 (9.47)	100.33 (13.02)	94.05 (13.97)
Single word reading t3	100.45 (18.62)	90.08 (19.54)	81.90 (14.88)	77.54 (13.00)
Phoneme deletion t5	110.66 (11.45)	107.92 (10.90)	99.43 (13.44)	101.17 (13.58)
Single word reading t5	107.10 (13.58)	103.00 (8.17)	93.33 (15.99)	94.71 (14.24)
Spelling t5	100.99 (13.96)	98.75 (10.56)	89.57 (11.36)	88.83 (14.77)
Prose reading comprehension t5	110.75 (10.06)	108.08 (4.83)	98.94 (11.66)	97.28 (6.60)

As predicted, the scores of the resolving group were similar to those of the TL group and both the emerging and persisting groups showed deficits on measures of literacy and related measures, as expected given their concurrent LI status. Literacy scores for the resolving group are not significantly different from those of the TL group on any measure and effect sizes are generally small. In contrast, the emerging and persisting LI groups perform worse than the TL and resolving groups and similarly to each other (effect sizes for the contrast between the resolving and emerging groups were large but not always significant). While all of the LI groups (resolving, emerging, persisting) have standard scores in the average range at t5 it is important to note that their scores are at least 10 standard score points below that of the TL group.

Finally, it is instructive to consider how many of the children within each group fulfilled criteria for ‘dyslexia’ at t5. In the broader Wellcome sample we have defined dyslexia using a criterion of 1 SD below the control mean on a composite measure comprising word reading and spelling scores; this corresponds to a standard score of 88. Using this criterion, 14% of the TL, 8% of the resolving (one child), 48% of the emerging and 41% of the persisting group are dyslexic.

## Discussion

This study tracked children from age 3½ through 5½ to 8 years, and considered their LI status across these three time points in order to classify LI trajectories. Of those with an LI at age 3½, 22% of cases had resolved by age 8 while 78% persisted. These figures differ from those reported by Bishop and Adams (1990) who found that 44% of LIs at age 4 resolved by age 8½ years but it is important to note that the present study recruited children at family risk of dyslexia as well as children with preschool LI. Also, as shown above, the cut‐off criterion for LI influences rates of language impairment such that when a more conservative criterion is used, fewer children show the pattern of resolving impairment although this has less effect on those classified with persisting language difficulties. For those children in our sample who demonstrated LI at some point, there were more boys in both of the groups who had LI at t1 and boys were significantly more likely to show the persisting trajectory. Importantly, there were more children at family risk of dyslexia in the emerging group, a finding which confirms the observation of Zambrana et al. (2014). Furthermore, boys and girls were represented equally in this group and the children were not socially disadvantaged. Together these findings are suggestive of a different etiology from that associated with preschool language impairment, possibly of genetic origin.

Our first aim was to characterize the language profiles of these different LI trajectories. Children with persisting LI always performed significantly below the TL group: they had marked, pervasive and sustained language difficulties, relative to their unaffected peers. Children with resolving LI, who were by definition, impaired on all language measures at age 3½ reached age‐expected levels at age 8 but it is noteworthy that their scores on language tasks were still lower than those of unaffected peers, particularly for expressive vocabulary. This replicates Bishop and Adams (1990) in demonstrating residual but subclinical effects of preschool LI in middle childhood; moreover, it highlights the possibility of ‘illusory recovery’ and the likelihood that some children with early LI may relapse in adolescence as language and literacy demands increase (cf. Snowling et al., 2000).

In terms of differentiating between pre‐school LIs that resolved versus persisted, children with resolving LI had significantly better comprehension and vocabulary knowledge at age 3½, but poorer grammar, than those with persisting LI. Furthermore, the resolving group had higher nonverbal IQ. This is compatible with other studies in showing that early LIs are more likely to resolve if they are less severe, and if they are accompanied by average nonverbal abilities (e.g., Bishop & Edmundson, 1987; Cole et al., 1995).

Turning to children with emerging LI, although they performed significantly below the TL children at age 3½, their standard scores across the language measures were in the average range. By age 8, their language scores were in the low‐average to below‐average range, falling significantly below those of the TL group and equaling those of the persisting group. It seems therefore that LIs that do not emerge until middle childhood will be difficult to detect (if relying on standard scores), confirming findings from preschool development (e.g., Dale et al., 2003; Henrichs et al., 2011); however, our findings suggest that a family risk of dyslexia (characterizing 76% of this group) might be a useful risk indicator.

Our second aim was to evaluate literacy outcomes for each LI trajectory. Consistent with the findings of Bishop et al., (op cit), the resolving LI group performed at the same level as the TL group on all literacy‐related measures and only one child in the group was identified as dyslexic at t5. Consistent with the critical age hypothesis, the language difficulties of children in this group were within the average range at t3, around the time of beginning formal reading instruction, and their emergent literacy was on course, although they still had relatively poor vocabulary and word recognition was somewhat delayed.

The emerging and persisting LI groups performed significantly worse than the TL group on all literacy‐related measures at ages 5½ and 8, consistent with previous findings that school‐age children with LI have concurrent literacy difficulties (e.g., Stothard et al., 1998). The emerging profile is particularly notable in that the late‐onset of their language difficulties observed at t3 coincides with the point of literacy instruction when literacy development was significantly delayed (average reading standard score of 82). While this profile raises the possibility of a ‘Matthew Effect’ for language and for nonverbal ability (after Stanovich, 1986), it is also consistent with mapping theories (e.g., Chiat, 2001) that propose that phonological processing impairments in preschool (typically considered risk factors for dyslexia) can have repercussions for the development of language, perhaps most especially vocabulary development.

Together our findings are sobering: regardless of whether an LI emerges early or late, if it is present in the early school years it has a negative impact on learning to read: 48% of the emerging and 41% of the persisting groups were identified as dyslexic at age 8. However, it is also important to be aware of possible differences in these two pathways to poor literacy. Our findings suggest that a broad range of adversities are associated with the persisting trajectory, including low SES (see Hoff, 2006 for discussion), and parent ratings not reported here suggest that a high proportion of these children have significant attention problems. In contrast, the emerging trajectory is primarily associated with family risk of dyslexia and the rates of social disadvantage and of attention problems are lower. The male liability is also lower for the emerging than for the persisting profiles. Future research should be directed toward understanding the differential predictors of emerging and persisting LIs.

Before concluding, we acknowledge limitations of this work. In particular, although we have attempted to improve reliability of measurement by using composite language measures we cannot circumvent the weaknesses of using arbitrary cut‐offs to create categorical variables (language groups) from continuous data. Moreover, the cut‐offs used to define LI affect the proportions of children classified as following the different trajectories. Further, it is possible that our findings depend upon the tests used; if we had included phonological processing skills in the diagnostic criteria some of the emerging group may have fulfilled criteria for LI at an earlier stage. Nonetheless, it remains common practice for children to be diagnosed with LI according to arbitrary cut‐points on language tests, and exploring between‐group differences can inform theoretical and clinical issues of importance. While we acknowledge the argument that resolution of LI over could be due to regression to the mean (Tomblin et al., 2003), we highlight the case of emerging LI in which language scores regress away from the mean over time.

## Conclusion

In this study, we characterized three trajectories of language impairment, and evaluated their literacy outcomes. Persisting LI, identified in preschool, was characterized as severe and pervasive, with relatively poor literacy outcomes. However, when preschool LI has resolved around the time of formal reading instruction, there is a generally good outcome for language and literacy, although with residual (subclinical) weaknesses in vocabulary which could still affect later outcomes. Possible protective factors are relative strengths in nonverbal ability and less extensive vocabulary difficulties at preschool (when compared with the persisting LI group). Finally, a third group of children had late‐emerging problems which were identified in middle childhood but not evident in preschool. Many of these children were at family risk of dyslexia and their language and literacy outcomes were as poor as those with persisting LI. Together these findings suggest there are at least two pathways to poor reading – one is the outcome of preschool LI, the other more specifically associated with family risk of dyslexia and associated with late‐emerging LI.

**Key points**

- We tracked children from ages 3½ through 5½ to 8 years and identified cases of resolving, persisting and emerging language impairment.
- Persisting LIs were more severe and pervasive than resolving LIs. Language and literacy outcomes were relatively poor for those with persisting LI, and relatively good for those with resolving LI.
- A significant minority of children with average pre‐school language abilities had LIs that emerged in middle childhood; these children are hard to identify early on but many are at family risk of dyslexia. We found support for the ‘critical age hypothesis’, but also identified children with average language at age 5½ who displayed significant reading difficulties later on.
- Many children with late‐emerging LIs fulfill criteria for dyslexia.
